# Evaluation of polypyrrole-modified bioelectrodes in a chemical absorption-bioelectrochemical reduction integrated system for NO removal

**DOI:** 10.1038/s41598-019-49610-2

**Published:** 2019-09-10

**Authors:** Tianjiao Guo, Chunyan Zhang, Jingkai Zhao, Cunhao Ma, Sujing Li, Wei Li

**Affiliations:** 1 Key Laboratory of Biomass Chemical Engineering of Ministry of Education, Institute of Industrial Ecology and Environment, College of Chemical and Biological Engineering, Zhejiang University (Yuquan Campus), Hangzhou, 310027 China; 2 College of Geography and Environmental Sciences, Zhejiang Normal University, Jinhua, 321004 China

**Keywords:** Environmental sciences, Pollution remediation

## Abstract

A Chemical absorption-bioelectrochemical reduction (CABER) system is based on Chemical absorption-biological reduction (CABR) system, which aims at NO removal and has been studied in many of our previous works. In this paper, we applied polypyrrole (PPy) on the electrode of bioelectrochemical reactor (BER) of CABER system, which induced a much higher current density in the cyclic voltammetry (CV) curve for the electrode itself and better NO removal rate in the system. In addition, a Microbial Electrolysis Cell (MEC) is constructed to study its strengthening mechanism. Results shows that PPy-MEC has a greater Faraday efficiency and higher reduction rate of Fe(III)EDTA and Fe(II)EDTA-NO in the solution when compared to original Carbon MEC, which confirms the advantage of PPy-modified electrode(s) in the CABER system. The results of this study are reported for illustration of potential of CABER technology and design of low-cost high-efficiency NO_*x*_ control equipment in the future.

## Introduction

Emission limitation of NO_*x*_, which are major precursors of PM2.5^1^, is an urgent task for thermal power plants in the field of air pollution control. A chemical absorption-bioelectrochemical reduction (CABER) integrated system, is based on the previous chemical absoprtion-biological reduction (CABR) system which is developed for highly efficient NO_*x*_ removal in the flue gas^2–6^, and uses biofilm electrode to enhance the system operation through strengthening the reduction process. Due to their advantages of low cost, high efficiency and non-secondary pollution, CABR and CABER have attracted researchers’ interests and are considered to be promising technologies for the removal of NO_*x*_ if compared to conventional methods in the past decades such as selective catalytic reduction (SCR)^7–9^, selective non-catalytic reduction (SNCR), absorption, or adsorption^10–12^.

A typical CABER technology consist of two major processes: First, NO (the primary component in NO_*x*_ in the flue gas) is absorbed through chelating Fe(II) ethylenediaminetetraacetic acid (EDTA) to form Fe(II)EDTA-NO:

$${\rm{Fe}}({\mathrm{II})\mathrm{EDTA}}^{2-}+{\rm{NO}}\leftrightarrow {\rm{Fe}}(\mathrm{II})\mathrm{EDTA}-{{\rm{NO}}}^{2-}$$

Meanwhile part of the Fe(II)EDTA is oxidized to Fe(III)EDTA by oxygen;

Then, Fe(II)EDTA-NO is reduced to Fe(II)EDTA and N_2_ in the biological electric reactor (BER) with electron donors such as glucose, electron from external voltage/current or even Fe(II)EDTA itself ^5,6,13^:

$$\mathrm{2Fe}(\mathrm{II})\mathrm{EDTA}-{{\rm{NO}}}^{2-}+4\,{e}^{-}+4{{\rm{H}}}^{+}\,\mathop{\longrightarrow }\limits^{{\rm{microorganism}}}\,{\mathrm{2Fe}(\mathrm{II})\mathrm{EDTA}}^{2-}+{{\rm{N}}}_{{\rm{2}}}+2{{\rm{H}}}_{{\rm{2}}}{\rm{O}}$$

$$\mathrm{2Fe}(\mathrm{II})\mathrm{EDTA}-{{\rm{NO}}}^{2-}+{\mathrm{2Fe}(\mathrm{II})\mathrm{EDTA}}^{2-}+{{\rm{4H}}}^{+}\,\mathop{\longrightarrow }\limits^{{\rm{microorganism}}}\,{\mathrm{4Fe}(\mathrm{III})\mathrm{EDTA}}^{-}+{{\rm{N}}}_{{\rm{2}}}+2{{\rm{H}}}_{{\rm{2}}}{\rm{O}}$$

And in the meantime, Fe(III)EDTA is also reduced into Fe(II)EDTA by microorganism.

Although there are debate on the primary electron donor, previous researches have reveal that the biofilm electrode reactor (BER), is the core part of the CABER technology; as the speed-limiting process, biological reduction of Fe(III)EDTA is the key that restrict the efficiency of the system^14,15^, and the electron pathway of Fe(III)EDTA bioreduction is a direct electron transfer process^16^. Thus as the electron transmission medium and room for microbes’ attachment and growth, modification of the electrode in BER would be crucial to the further development of the CABER technology.

On the other side, conductive polymers, which refers to organic polymers that conduct electricity, has been another research focus due to their high processability^17^. The electrochemical properties of conductive polymers originate from their conjugated structures, and can be tuned into metallic conductivity materials or semiconductors with organic synthesis^18^ and dispersion techniques^19^. According to the properties of polymers, electronic donors or acceptors are doped, and electrons are injected or removed into the molecular chains of conductive polymers, so as to optimize the electronic conductivity of conductive polymers. And they have been successfully used in fields of battery cathodes^20^, microelectronics^21^, nonlinear optics^22^, and sensors^23^, etc.

In this vein, we applied polypyrrole (PPy), a conductive polymer that has good stability and excellent biocompatibility^24^ on to the surface of carbon electrode in a BER and tested its performance in a CABER system. Previous related researches about polymer enhanced electrode reveals that they have a wide application as sensors in determinations of heavy metal ions^25^, morphine^26^, glucose^27^ acetaminophen^28^, nitrite^29^, ascorbic acid, dopamine, uric acid and xanthine^30^, etc. As to PPy doped electrode specifically, researches shows that it has an improved affect in power output in membrane fuel cells^31^ and a greater electrochemical signal in sensors^32^. However, there isn’t any work as far as we know that shows its application in the field of air pollution control such as NO_*x*_ removal in this work. In this study, we report result of preparation of the modified electrode itself, and make it into working electrodes and tested their performance in a CABER system.

As to the mechanism study of electron trasnfer of biocathode in such bioelectrochemical system, our previous work^16^ has already showed that bacteria has the ability of direct electron uptake from the electrode via physical contact and related bacterial nanowires has been observed. Thus the strengthening mechanism in this work is mainly focused on the characterization and morphology of the electrode and macro performance of a Microbial Electrolysis Cell (MEC) that constructed from the modified electrodes and comparison to a traditional carbon MEC (C-MEC). It is hoped that this work can provide more evidence of potential of the CABER technology and insights in design of new low-cost air pollution control equipment which is still a vacancy in the practical application of high-efficient NO removal.

## Results and Discussion

### Electrochemical polymerization of pyrrole on the electrode and characterization of the produced film

**Figure 1:**
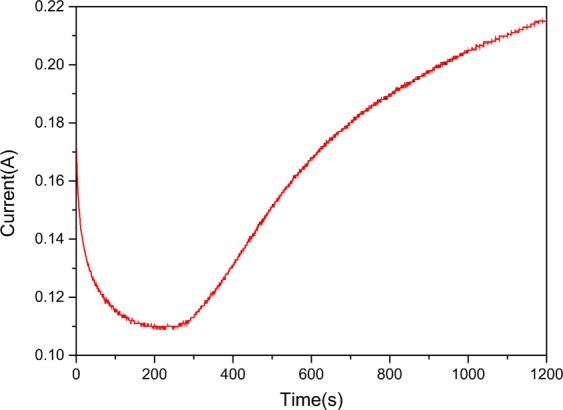
Electropolymerization of pyrrole using potentiostatic method.

Figure 1 shows that the formation of polypyrrole film is a point-to-surface process and follows the traditional three-step process: nucleation, growth and expansion. 0–200 s is the initial stage of electrochemical polymerization process, and the polymerization current decreases. This stage is short, and the formation of polypyrrole film is not uniform, which affects the conductivity of the electrode. After 200 s, it enters the later stage of polymerization: because sodium p-toluene sulfonate (NaPTS) is gradually doped in the polymer, sulfonic anions were filled into the polymer chain. As the advance of the dispersion process, the electron mobility in the polymer chain were improved; the conductivity was enhanced, and the current was gradually increased, until a uniform and stable polypyrrole film was formed. The final conductivity of the PPy modified electrode was 1.3 times that of the traditional graphite electrode. Thus the electronic transmission capability of the electrode is improved.

**Figure 2:**
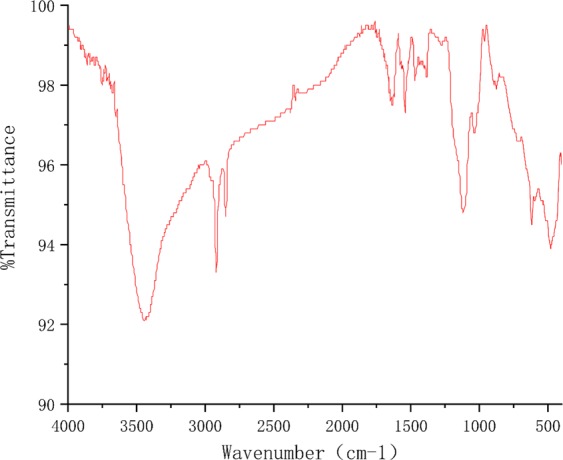
FT-IR image of the powder of the produced film

**Figure 3:**
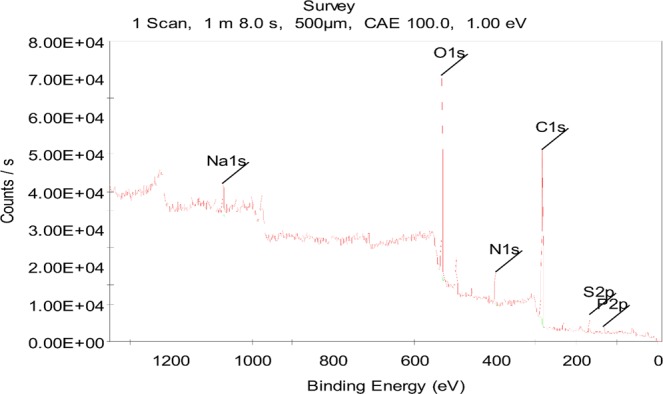
XPS image of the powder of the produced film.

The produced polypyrole film is then measured by a outside micrometer for its thickness, which leads to a value of about 0.43 mm (Table S1 in Supplementary Information). Also, FT-IR image of the scraped powder on the electrode surface (Fig. 2) shows that the characteristic bands of the produced PPy film are consistent with those in^33,34^: the peak at 3450 cm^−1^ can be assigned to the N-H streching mode^33^, some other observed peaks are the pyrrole ring fundamental vibration at 1543 and 1467 cm^−1^, the C-H in-plane vibration at 1380 and 1034 cm^−1^, and the C-N stretching vibration at near 1120 cm^−1^ ^34^. The results indicate that the molecular structure of the PPy chain is almost identical to that those in the literature. In addition, a wide scan XPS of the scraped powder detected Na, O, N, C, S, P six elements (Fig. 3), whose atomic ratios are: 2.39:25.55:7.56:59.85:3.15:2.49, indicating substances as graphene oxide may also existed. Nevertheless, the result in Fig. 3 is consistent with that in^35^.

### Electrochemical performance and morphology analysis of polypyrrole modified electrode

**Figure 4:**
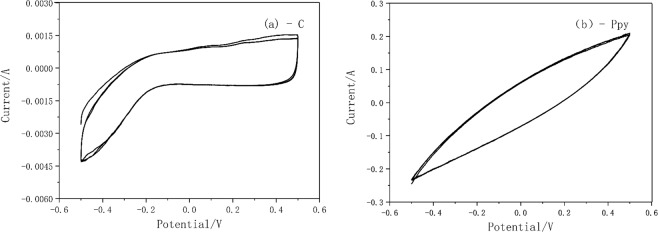
Cyclic voltammetry curves of C/PPy electrodes. (a. C; b. PPy; 0.1 M H_2_SO_4_; 50 mV/S; −0.5 V-0.5 V).

The electrochemical characteristics of Polypyrrole modified electrode was then investigated by cyclic voltammetry with 0.1 M H_2_SO_4_ as supporting solution. The electrochemical stability was determined by multiple scanning, as shown in the CV curve in Fig. 4, which also reveal that, at the same voltage the current of PPy modified electrode is about 100 times of that of original graphite electrode. The output current of PPy modified electrode is high and its properties are stable after several scans. It can be seen that PPy modified electrode exhibits excellent electrochemical property and has a great potential in improving microbial reduction process.

**Figure 5:**
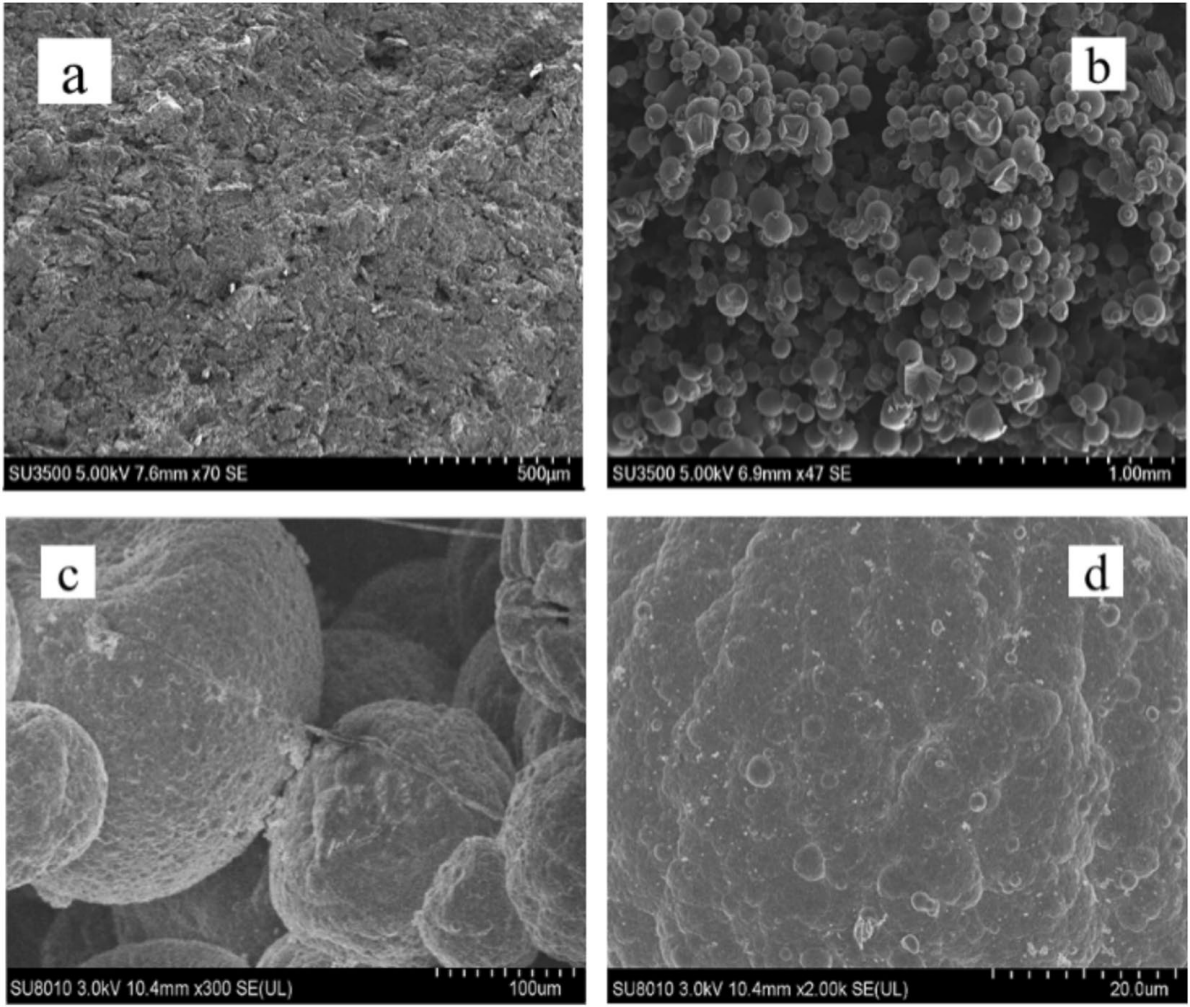
SEM graphs of PPy/C electrodes. (Py = 0.1 M; NaPTS = 0.075 M; H_2_SO_4_ = 0.1 M; (**a**) C electrode; (**b**–**d**) PPy electrode).

The morphology of Polypyrrole Modified electrode and traditional graphite electrode were then observed under scanning electron microscope (SEM) as shown in Fig. 5. The traditional graphite electrode surface is relatively flat, while the PPy modified electrode surface has typical PPy “microsphere” structure (diameter is about 10–100 μm), and each single microsphere surface protrudes “cauliflower” structures. Therefore, the PPy modified electrode has a larger specific surface area, which can provide more sites for microbial growth and bioreduction process.

### Start-up of a CABER System with PPy modified electrode

The PPy modified electrode was then used for the working electrodes in a CABER system, as described in Section: Experimental. And the details of the start-up process can be seen at Fig. S1 and related section in Supplementary Information. In this process we found that the treatment load and iron reduction performance of the system were both higher than those of CABER system without electrode modification^36^. After 25 days of operation, the reduction efficiency fluctuated slightly but remained stable to the last day. This shows that the microorganisms on the surface of PPy modified working electrodes had successfully formed a film, and the start-up process of a CABER system was completed.

**Figure 6:**
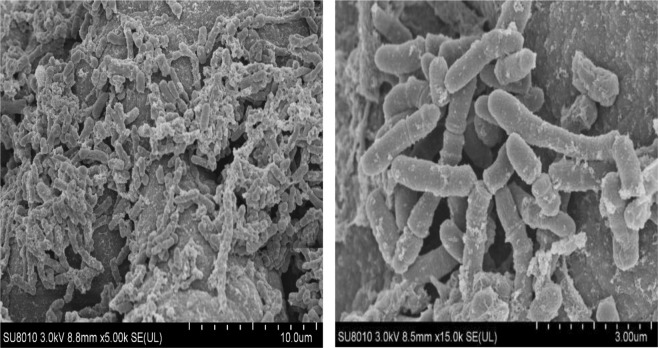
Surface of PPy modified working electrodes after film-forming process

The morphology of the biofilm on the surface of PPy modified working electrodes after the start-up of the CABER system is shown in Fig. 6. Mixed biofilms were formed on the surface of the particles, and typical rod-shaped iron-reducing bacteria were observed.

### PPy modified electrode constructed microbial electrolysis cell

Since it was found that the new biofilm electrode reactor with PPy modified electrodes had better removal effect of NO in the start-up process, in order to explore the enhancement effect of modified electrodes in detail, the PPy modified electrodes were taken out, and constructed into a smaller microbial electrolysis cell (PPy-MEC) for further investigation of the bioreduction process of Fe(III)EDTA, which is the speed-limiting step in the system, with comparison of traditional carbon electrode particles constructed microbial electrolysis cell (C-MEC).

Considering the two main operation methods of PPy-MEC: constant potential and constant current, various potential and current conditions were performed for test purposes. And test results can be seen at Fig. S2 in the Supplementary Information. Among them, −300 mV and 500 mg of glucose maintain a highest Fe(II)EDTA concentration. Thus −300 mV is selected for the constant potential method. Also, according to the CV curve of Fig. 5, the output current of 0.02 A under the optimal potential is also selected for constant current method to run MECs.

#### Bioelectrochemical reduction of Fe(III) EDTA

**Figure 7:**
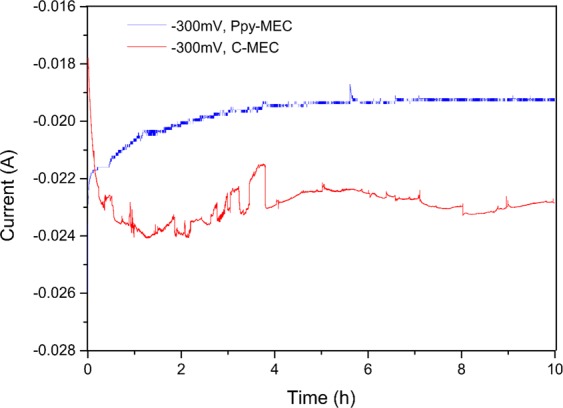
Current comparison of PPy-MEC and C-MEC at −300 mV potential. (potential = −300 mV; Fe(III)EDTA = 10 mM; V_L_ = 300 ml; pH = 7.0 ± 0.2; G_0_ = 0 mg).

**Figure 8:**
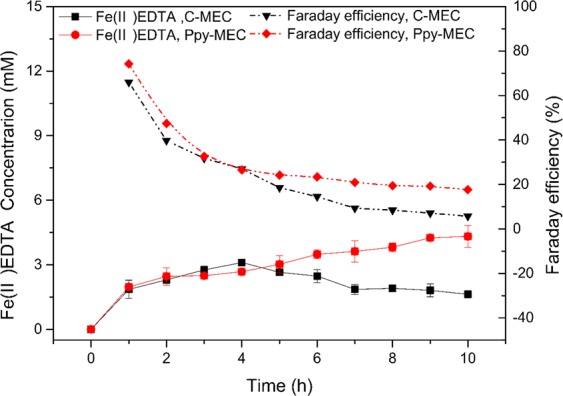
Fe(III) reduction and faraday efficiency at PPy-MEC and C-MEC. (potential = −300 mV; Fe(III)EDTA = 10 mM; V_L_ = 300 ml; pH = 7.0 ± 0.2; G_0_ = 0 mg).

In a PPy-MEC which is built based on Polypyrrole modified electrodes (described in Section: Experimental), the potential of the electrode was controlled at −300 mV, and the bioelectrochemical reduction process of Fe(III)EDTA was investigated without adding carbon source. Figure 7 shows the dynamic response of current in PPy-MEC and C-MEC under constant potential. In the process, the corresponding current of PPy-MEC is lower than that of C-MEC. This is because the specific surface area of PPy is better than that of graphite electrode, so it loads more microorganisms on its surface. And its biological affinity made the expansion of biofilm increases PPy’s electrochemical resistance. With the advancement of bio-reduction process, the trends of both currents are similar, and both of them decrease gradually; because the concentration of reactant Fe (III) in solution decreases with the reduction process, so the rate of electron transport and transfer decreases gradually, and it finally reaches a stable state. However, the reduction rate of Fe (III) EDTA of PPy modified electrodes is higher than that of conventional graphite electrodes, as it can be seen in Fig. 8. In addition, by investigating the Faraday efficiencies of the two systems under the same conditions, we can explore the reasons why PPy modified electrodes enhance bioreduction process in the CABER system from the level of electronic utilization. Figure 8 shows that the Faraday efficiency in PPy-MEC decreases from 74.24% to 17.64%, which is 1–3 times higher than that in C-MEC (65.95–5.72%). Therefore, the electrochemical performance of PPy modified electrode is better than that of traditional graphite electrode, and the electron transfer efficiency can be the internal cause of strengthening the reduction process.

**Figure 9:**
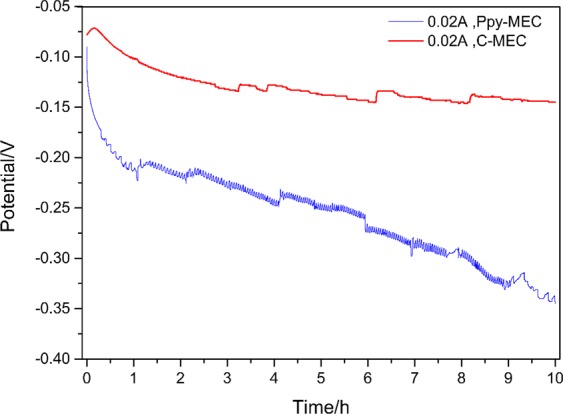
Potential comparison of PPy-MEC and C-MEC at −0.02 A. (I = −0.02 A; Fe(III)EDTA = 10 mM; V_L_ = 300 ml; pH = 7.0 ± 0.2; G_0_ = 0 mg).

**Figure 10:**
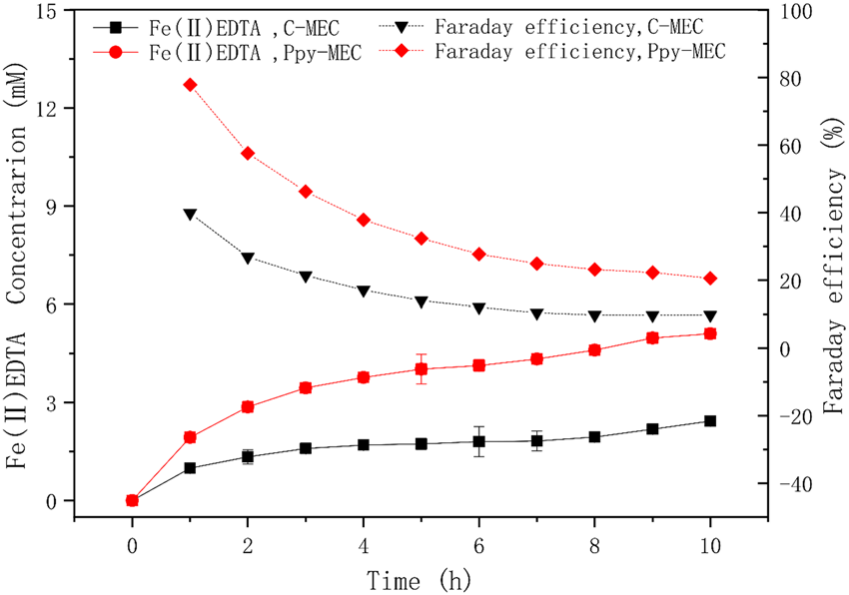
Fe(III) reduction and Faraday efficiency in PPy-MEC and C-MEC. (I = −0.02 A; Fe(III)EDTA = 10 mM; V_L_ = 300 ml; pH = 7.0 ± 0.2; G_0_ = 0 mg)

The bioreduction process of Fe (III) EDTA was also investigated through constant current method which holds working current of the MEC at −0.02 A, which corresponds to a current density of −0.885 A/m^2^.The potential changes in MEC without additional carbon source were shown in Fig. 9. With the reduction of Fe (III) EDTA, the concentration of the reactant decreases. In order to maintain the stability of the electrochemical system, the potential of the electrode decreases gradually. In this process, the average bioreduction rate of Fe (III) in the modified electrode system is 0.511 mmol h^−1^, while that of Fe (III) in the non-modified system is only 0.243 mmol h^−1^. On the other hand, the average reduction rate of trivalent iron with constant current method is higher than that with constant potential method. This is due to the constant current method provides a relatively stable electron output rate, but the defect of this method is that the potential may be lower than the potential of hydrogen evolution in the later stage, resulting in system fluctuation^16^. In this process, as shown in Fig. 10, the Faraday efficiency of PPy-MEC decreased from 77.8% to 20.53%, while that of C-MEC decreased from 39.8% to 9.77% under the same conditions, which was only one half of that of the PPy-MEC.

#### Bioelectrochemical reduction of Fe(II) EDTA-NO

**Figure 11:**
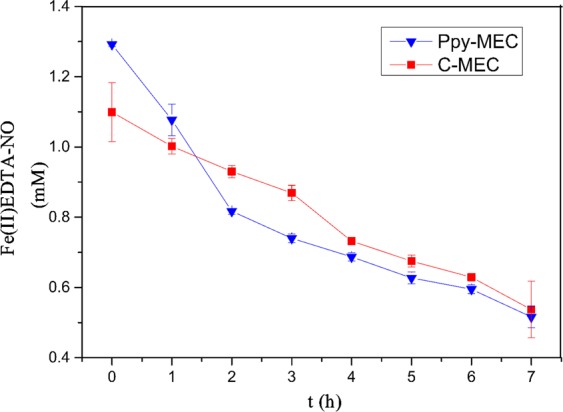
Fe(II)EDTA-NO reduction in PPy-MEC and C-MEC. (potential = −300 mV; Fe(III)EDTA = 10 mM; V_L_ = 300 ml; pH = 7.0 ± 0.2; G_0_ = 0 mg).

Under the condition of constant potential (−300 mV), microbial electrolysis cells were operated steadily, and the reduction process of Fe(II) EDTA-NO in PPy-MEC and C-MEC was investigated intuitively. The experimental results were shown in Fig. 11. Compared with the system without electrode modification, the reduction rate of Fe(II)EDTA-NO in PPy-MEC system is 1.4 times higher than that in C-MEC system, and the reduction process is stable. PPy modified electrodes improve the electron transport efficiency, thus improving the electron utilization efficiency of microorganisms, thereby enhancing the denitrification performance of the system.

## Experimental

### Chemicals

Preparation of Fe(III) EDTA Solution: A 100 mM Fe(III) EDTA solution was prepared with equal molar Na_2_EDTA and FeCl_3_·6H_2_O.

Preparation of Fe(II) EDTA Solution: A 30 mM Fe(II) EDTA solution was prepared with equal molar Na_2_EDTA and FeSO_4_·7H_2_O.

Preparation of Fe(II) EDTA-NO Solution: A 30 mM Fe(II) EDTA solution was equally transferred into two absorption bottles, and N_2_ was used as protective gas to pass into NO gas to saturate the solution. The concentration of Fe(II) EDTA-NO was determined before use.

Na_2_EDTA·2H_2_O (99%), FeCl_3_·6H_2_O (99%), D-glucose (99.5%) and other chemicals were applied in analytical reagent grade without further purification, which were purchased from Sinopharm Chemical Reagent Co. (Shanghai, China). The compositions of simulated flue gas were supplied by Zhejiang Jingong Gas Co. (Hangzhou, China): N_2_ (99.999%), CO_2_ (99.999%), O_2_ (99.999%), and NO (5 vol% in N_2_).

### Medium and organism

The nutrient medium contained (g/L): trace elements, 0.002; CaCl_2_, 0.02; Na_2_SO_3_, 0.07; MgCl_2_, 0.1; KH_2_PO_4_, 0.3; and glucose, 1. The trace elements included (g/L): H_3_BO_4_, 0.014; ZnCl_2_, 0.1; NiCl_2_∙6H2O, 0.19; CoCl_2_, 0.24; CuSO_4_∙5H_2_O, 0.25; and MnCl_2_∙4H_2_O, 0.99.

The bacteria that used for denitrification and iron-reducing were obtained from our previous work^5,13^.

## Analytical Methods

Fe(II) EDTA concentration determination: using o-phenanthroline spectrophotometry; 0.5 mL HCl(2 M) was added to the 50 mL volumetric flask, followed by 0.5 mL samples, 2.5 mL o-phenanthroline(0.15%) and 5 mL CH_3_COONa(1 M). The solution was then calibrated to 50 mL and bathed in water at 30 C. After 20 minutes, the absorbance (510 nm) was measured, and the standard curve of Fe concentration was revised before measurement.

Total iron (FeEDTA) concentration determination requires same HCl and the sample to be added, followed by 1 mL hydroxylamine hydrochloride (1%) solution, resting for 5 minutes, waiting for the Fe(III) reduction occurs and then repeated the same above steps for spectrophotometry measurement. The concentration of Fe (III) is the difference between total iron and Fe (II) ion.

Fe(II) EDTA-NO concerntration determination: using spectrophotometry; 5 mL samples were filtered by 0.22 um filter membrane, and the absorbance at 420 nm was read.

FT-IR was conducted with a Gas Chromatography-Fourier Transform Infrared Spectrometer (SGE/Agilent 6890/Nicolet 5700), and XPS was performed by a Photoelectron Spectrometer (Thermo Scientific ESCALAB 250Xi).

Electrode morphology was observed by SEM, and cyclic voltammetry (CV) was used, and the dynamic changes of potential and current of the new electrode in MEC were monitored so as to investigate its electrochemical properties. For convenience of measurement, the potential of the electrode presented in this study is relative to the standard hydrogen electrode (SHE).

All concentration data were replicates of at least three readings, with results represented with mean value and plusminus one standard deviation (±σ).

### Devices

**Figure 12:**
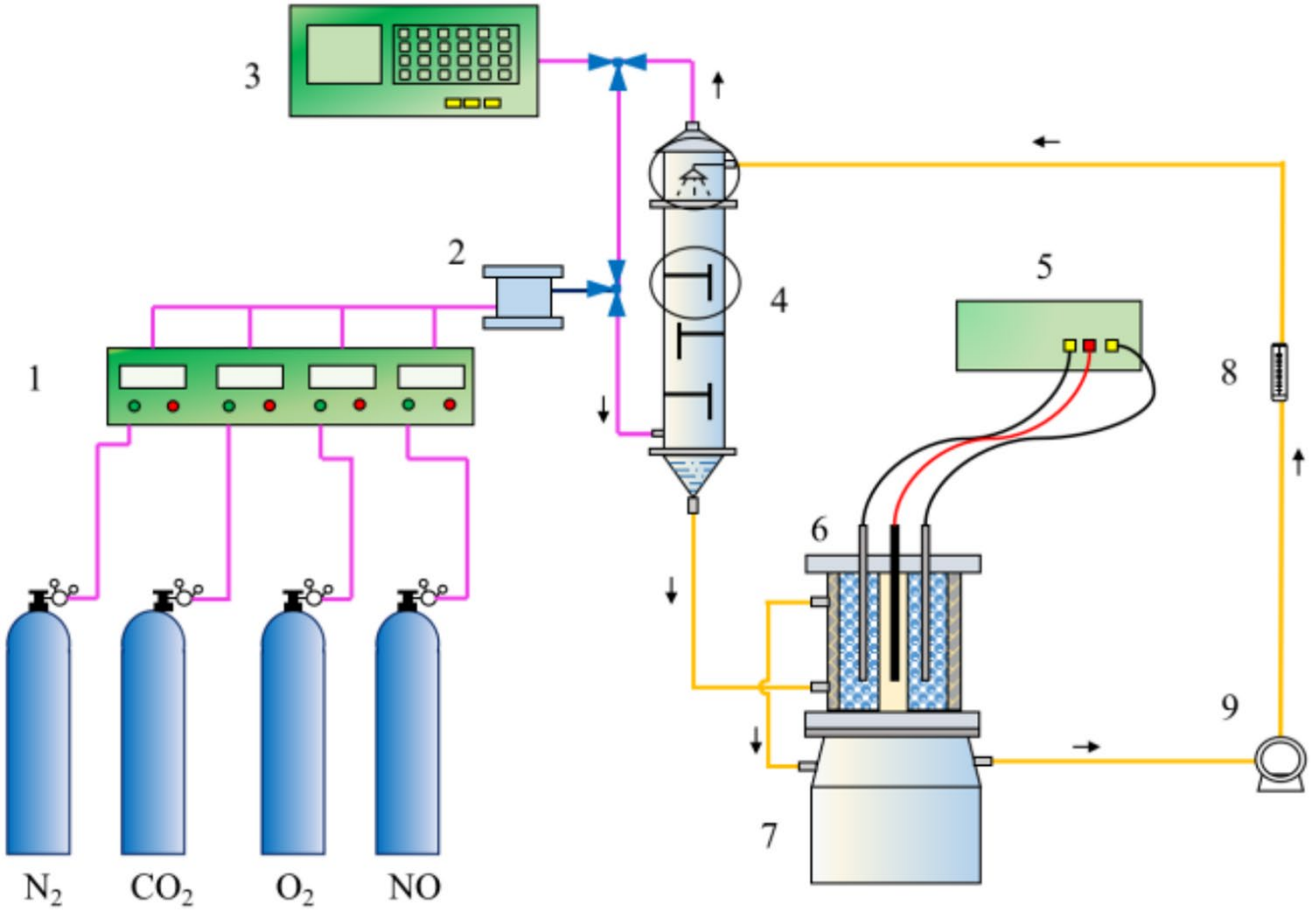
Schematic of the lab-scale CABER system. (1ˎ8. mass flow controller; 2.gas maxing chamber; 3. NO_*x*_ analyzer; 4. sieve tray tower; 5. DC power; 6. BER; 7. water bath; 9. pump).

The structure of laboratory scale CABER device is shown in Fig. 12. The sieve tray tower has three baffles with an inner diameter of 0.04 m and an effective volume of 0.57 L. NO, O_2_, N_2_ and CO_2_ are formed into simulated flue gas through gas mixing chamber, and the absorption process is completed from the lower part of sieve tray tower. BER is made of plexiglass with inner diameter of 0.07 m, outer inner diameter of 0.12 m, and it has a total reactor volume of 1.9 L and effective volume of 1.24 L. The carbon rod in the center of the anode chamber is 10 mm in diameter, and the same carbon rod in the left and right of the cathode chamber serves as the connecting electrode. The cathode chamber is filled with new working electrode particles (Φ 6 mm × 10 mm), which is the main area of microbial film forming. The constant temperature water bath jacket is controlled at 50 °C. The volume of the tank below is 2 L.

**Figure 13:**
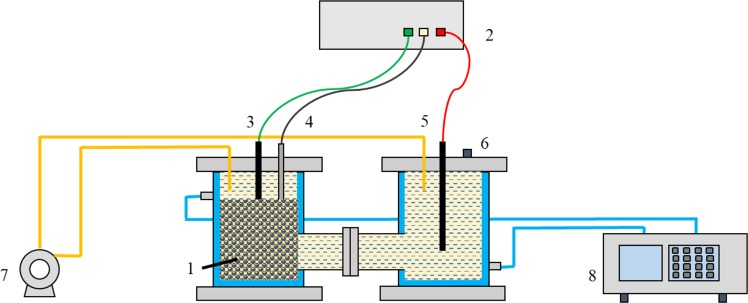
Schematic of the microbial electrolysis cell. (1. modified microbial working electrodes; 2. electrochemical workstation; 3. modified connecting electrode; 4. reference electrode (Ag/AgCl); 5. counter electrode(graphite); 6. injection/sampling port; 7. pump; 8. water bath).

The main body of the microbial electrochemical reduction system is a two-chamber electrolytic cell reactor, as shown in Fig. 13. The temperature of the circulating water bath is controlled at 50 C. The circulating pump makes the culture medium between the two rooms circulate and speeds up the mass transfer efficiency. The working electrodes made of modified electrodes were filled in the left cell, and a graphite rod were used as connecting electrodes, the reference electrodes were Ag/AgCl electrodes, and the external electrochemical workstations were connected to provide potential driving force.

## Experimental Procedures

The electrochemical polymerization of pyrrole was carried out in a three-electrode electrolytic cell by potentiostatic method. The working electrode is graphite electrode (d = 6 mm), the auxiliary electrode is platinum wire, and the reference electrode is silver/silver chloride electrode (vs. Ag/AgCl). The polymerization process is controlled by an electrochemical workstation with a constant voltage of 0.9 V. In the process, NaPTS is selected as the dopant. With 0.1 M pyrrole (Py) monomer and 0.075 M NaPTS, the electrolyte was supported by 0.1 M H_2_SO_4_ solution (pH = 2.28), which ensured that the electrochemical polymerization maintained a low pH, and the potentiostatic polymerization lasted 1200 seconds at 30 C. Attention should be paid that nitrogen is supplied for oxygen removal for 10 minutes before the experiment starts; graphite electrodes are polished before use: ultrapure water, absolute ethanol and ultrapure water are used consecutively for ultrasonic processes to clean the surface, each lasts for 5 minutes. The prepared PPy modified electrode was then dried and reserved for later use. The electrochemical properties were tested and the surface morphology through SEM was observed.

The PPy modified electrode was prepared into working electrode particles (d = 6 mm, H = 1.2 cm) and filled into the cathode chamber of the BER. And the constructed PPy-BER was coupled with the sieve tray tower and the whole CABER device is as shown in Fig. 12. 3 L absorbent liquid was prepared with nutrient medium (300 mL), Fe(III)EDTA solution (300 mL), NaHCO_3_ buffer solution (300 mL), trace elements (5 mL) and mixed bacterial solution. The circulating liquid flow rate was 10 L h^−1^. The simulated flue gas composition is NO (450 ppm), O_2_, CO_2_, N_2_, with a total gas flow rate of 1 L min^−1^. In the initial stage, 1 L fresh culture medium containing bacterial liquid was replaced every day. According to the operating conditions of the integrated system, oxygen concentration was gradually increased, glucose was continuously added, and iron reduction process and nitrogen oxide removal effect were monitored. The microbial membrane in the electrode bioreactor was observed by SEM after the integrated system was able to operate stably with an applied current of 0.04 A.

In order to investigate the bioreduction enhancement of modified PPy working electrodes, a miniature polypyrrole microbial electrolysis cell (PPy-MEC) was constructed as shown in Fig. 13, in which the total particle size of the electrodes was 27.13 cm^3^. The PPy working electrodes were removed from the CABER system and placed in the PPy-MEC working room. The electrochemical workstation provided additional potential to start the PPy-MEC for preliminary cultivation. After stable operation of PPy-MEC, the performance of PPy working electrodes were investigated by sequential batch experiments, including: (1) Enhancement of biological reduction of Fe(III)EDTA alone: 300 mL Fe(III) EDTA(10 mM) solution was added to MEC after nitrogen supply for oxygen removal. Controlling cathodic potential, PPy-MEC operates steadily for 10 hours under constant potential condition to monitor the reduction of Fe(III) EDTA during this process; controlling cathodic current to monitor the reduction process of Fe(III)EDTA under constant current condition; (2) Enhancement of biological reduction of Fe(II) EDTA-NO alone: controlling cathodic potential to investigate the reduction process of chelated NO in this process. In the above three experimental processes, MEC operates in a carbon-free state for 12 hours before the experiment, ensuring that the original carbon source of the system is completely consumed by microorganisms, and preventing the interference of carbon source as an electronic donor.

## Conclusions

In this study, PPy modified electrodes was prepared and tested alone, and applied as working electrodes in a CABER system and in a microbial electrosis cell, with comparison to (a) traditional graphite electrode(s). Results shows that the PPy modified electrode has higher electron transport and migration ability, and can enhance the performance of CABER system. From the perspective of electron transport, the reason for reduction enhancement is mainly from the characterization and morphology of the modified electrode, which result in a better electrochemcial property and thus a higher Faraday efficiency and improved reduction process of both Fe(III)EDTA and Fe(II)EDTA-NO. A detail procedure of all experiments described in this study is also offered for future references.

## Supplementary information

Supplementary Information
