# Supplementary material for: Evaluation of polypyrrole-modified bioelectrodes in a chemical absorption-bioelectrochemical reduction integrated system for NO removal
Source: Sci Rep. 2019 Sep 10;9:13030. doi: 10.1038/s41598-019-49610-2 (PMC6737099; doi:10.1038/s41598-019-49610-2)
Supplement: Supplementary file 1 — Supplementary Information [file 41598_2019_49610_MOESM1_ESM.docx]

**Appendix: Supplementary information**

**Evaluation of polypyrrole-modified bioelectrodes in a chemical absorption-bioelectrochemical reduction integrated system for NO removal**

**Tianjiao Guo**1,+**,Chunyan Zhang**1,+**, Jingkai Zhao**2**, Cunhao Ma**1**, Sujing Li**1**, Wei Li**1,*

1 Key Laboratory of Biomass Chemical Engineering of Ministry of Education, Institute of Industrial Ecology and Environment, College of Chemical and Biological Engineering, Zhejiang University (Yuquan Campus), Hangzhou, 310027, China

2 College of Geography and Environmental Sciences, Zhejiang Normal University, Jinhua 321004, China

* Correspondence and requests for materials should be addressed to W.L. (email: w_li@zju.edu.cn)

+these authors contributed equally to this work

4 pages

**Measurement of thickness of the produced PPy film on the electrodes**

10 samples (including five coated one and five original one) of the electrodes were taken out and measured for the diameter, as shown in Table S1.

Table S1: Samples measured for thickness of the electrodes

|  | Sample1/6 | Sample 2/7 | Sample 3/8 | Sample 4/9 | Sample5/10 | Average |
| --- | --- | --- | --- | --- | --- | --- |
| Without film  (mm) | 6.14 | 5.87 | 6.08 | 6.07 | 6.14 | 6.06±0.11 |
| With film  (mm) | 6.71 | 6.78 | 6.83 | 7.19 | 7.12 | 6.93±0.21 |

**Start-up of CABER system with PPy modifed electrodes**

The start-up process of the system was mainly divided into four stages, as shown in Figure S1. In the first stage, the oxygen concentration was 0%: this is because of the toxic effect of oxygen on microorganisms, and microbial activity was maintained under anaerobic conditions to achieve the initial growth of microorganisms. When the concentration of Fe(II) EDTA gradually increased to 4 mM and the denitrification efficiency of the system reached 90%, the denitrification bacteria and iron reducing bacteria started to show good reductive activity. In the second stage, the oxygen concentration was increased to 3%: after oxygen was introduced into the simulated flue gas, the denitrification efficiency decreased due to the impact of oxygen. After 5 days of culture adaptation, the denitrification efficiency could be maintained at about 88%. In the third stage, when the oxygen concentration increases to 6%, the denitrification efficiency decreased from 88% to the lowest 72.9%, and gradually increased to 85%. This indicated that the microorganisms in the system had tolerance to oxygen at this time. Finally, the oxygen concentration in the fourth stage reached 9%: because the system had the ability to resist oxygen shock, the denitrification efficiency did not fluctuate greatly in the last two stages. It can also be seen that after 36 hours of running in the fourth stage, the denitrification efficiency increased to 90%, and the concentration of Fe(II) EDTA increased gradually to 3.854 mM, and the denitrification efficiency was stable at about 88%.

**Figure S1**. Start-up of the PPy-CABER system

(NO=450 ppm; I=0.04 A; gas flow rate=1 L min^-1^；liquid flow rate=10 L h^-1^; pH = 7.0 ± 0.2)

**Tests for various potential and current conditions to run the MEC**

**Figure S2**. Effect of different potentials and carbon source amounts to PPy-MEC

(Fe(III)EDTA_0_=10mM；V_L_ = 300ml；pH = 7.0 ± 0.2)
